# High glucose-induced intestinal epithelial barrier damage is aggravated by syndecan-1 destruction and heparanase overexpression

**DOI:** 10.1111/jcmm.12523

**Published:** 2015-02-20

**Authors:** Qing Qing, Shaoheng Zhang, Ye Chen, Runhua Li, Hua Mao, Qikui Chen

**Affiliations:** a Department of Gastroenterology, Sun Yat-sen Memorial Hospital, Sun Yat-sen UniversityGuangzhou, China; b Department of Gastroenterology, The Third Affiliated Hospital of Guangzhou Medical UniversityGuangzhou, China; c Department of Gastroenterology, Zhujiang Hospital, Southern Medical UniversityGuangzhou, China; d Department of Gastroenterology, Nanfang Hospital, Southern Medical University, Guangdong Provincial Key Laboratory of GastroenterologyGuangzhou, China

**Keywords:** high glucose, diabetes, syndecan-1, heparanase, intestinal epithelial barrier

## Abstract

Syndecan-1 (Sdc1) and its endo-beta-d-glucuronidase heparanase (HPSE) are implicated in maintenance of intestinal epithelial barrier (IEB), but their alterations and roles in high-glucose/hyperglycaemia (HG) conditions have not been fully investigated. This study aimed to determine the expression pattern, the possible regulation mechanism of Sdc1 and HPSE in HG conditions, and their potential effects on IEB. Therefore, diabetic mice/cell models were developed, and tissue/serum samples, cell lysate and culture supernatants were harvested. The expression of Sdc1 and HPSE in control, HG and designated interventions groups were detected. Phosphorylations of mitogen-activated protein kinase signalling pathway (MAPK), the expressions of Occludin and ZO-1, and the levels of transepithelial electrical resistance (TEER) were measured and monitored. The results showed that in HG conditions, intestinal tissue and cellular Sdc1 were significantly decreased, but the expression of HPSE, and soluble Sdc1 in serum and culture supernatants were remarkably increased. Such alterations of Sdc1 and HPSE were associated with solely p38 MAPK activation, and were correlated with the reductions of Occludin, ZO-1 and TEER. Heparin (Sdc1 analogue) and SB203580 (a p38 MAPK inhibitor), instead of insulin, alleviated Sdc1 destruction and HPSE overexpression, and effectively prevented against the reductions of tight junctions and the abnormality of intestinal permeability in HG conditions. In conclusion, we confirm the unique alterations of Sdc1 and HPSE in HG conditions, and found their interactions with p38 MAPK activation and IEB. These indicate that Sdc1/HPSE modulation can be viewed as an important complementary treatment for relieving HG-induced gastrointestinal damage.

## Introduction

Diabetes mellitus (DM) is a chronic disease requiring lifelong medical attention. With hundreds of millions suffering worldwide and a rapidly rising incidence, DM poses a great burden on healthcare systems 1,2. It is worth to note that, besides relevant cardiovascular diseases, neuropathy, and kidney failure, gastrointestinal complications also occur frequently in diabetic patients 3. They could be manifested by complaints (such as gastroparesis, constipation and diarrhoea), or lead to morphological and functional changes of the gastrointestinal tract (raise in mucosal surface area, intestinal weight and number of goblet cells per villus, increased intestinal permeability, *etc*.) 3–5. Nowadays, however, it is too early to claim a complete insight of the inherent links between DM and diabetes-associated enteropathy. Further studies investigating the pathogenetic and regulation/control factors are urgently needed.

Syndecan-1 (Sdc1), a predominant member of type I transmembrane heparan sulphate proteoglycans, plays important roles in inflammation, wound healing and tumour progression 6–8. Its structure mainly consists of a short conserved cytoplasmic and transmembrane domain, and a long, variable ectodomain carrying heparan sulphate (HS) chains. Membrane-bound Sdc1 can constitutively shed in a soluble form to extracellular and systemic circulation. It is accelerated by a variety of extracellular stimuli and matrix proteinase, such as heparanase (HPSE, an endo-beta-d-glucuronidase) 9–11. Recent studies indicate that the interplay between Sdc1 and HPSE (also named as ‘Sdc1–HPSE axis’) carries broad biological connotations in formation and maintenance of intestinal barrier 12,13. Studies have also suggested that intestinal specific loss of Sdc1 and abnormal HPSE might wreck the natural barrier, causing far more susceptibility to protein-loss enteropathy and bacterial translocation 14,15. As the diabetic state and the dysregulation of Sdc1/HPSE both lead to the gastrointestinal disorders, we wonder whether there are any certain interactions between them. However, the possible interactions have been rarely investigated, and the potential roles of Sdc1/HPSE in diabetic enteropathy have not been explored.

Therefore, we developed diabetic mice/cell models, applied biological experiments to study the alterations of Sdc1 and HPSE in high-glucose/hyperglycaemia (HG) conditions, and then investigated the subsequent changes of barrier function of intestinal epithelium and the possible regulation mechanism. We reported here that after HG stimulation, the dramatic Sdc1 destruction with synchronous HPSE elevation were presented and were correlated with abnormities of intestinal permeability, tight junctions and the activation of p38 mitogen-activated protein kinase (MAPK) signalling pathway. Heparin (which mimics Sdc1 function) and inhibitors targeting p38 MAPK pathway, instead of insulin, effectively reduced the alterations of Sdc1 and HPSE, and improve the impaired barrier function. These results suggest a potential mechanism of diabetic enteropathy, which is depending remarkably on Sdc1 and HPSE, and indicate a feasible option for future therapeutic strategy.

## Materials and methods

### Chemicals and antibodies

d-glucose, l-glucose and insulin were purchased from Sigma-Aldrich (St. Louis, MO, USA). Sdc1 polyclonal antibody (sc-7099) and HPSE monoclonal antibody (mAb, sc-25826) were purchased from Santa Cruz Biotechnology (Dallas, TX, USA), and soluble Sdc1 ELISA kits and heparanase ELISA kits were purchased from Diaclone (Besançon, France) and Cusabio (Wuhan, China) respectively. Rabbit polyclonal anti-occludin and anti-ZO-1 antibodies were obtained from Invitrogen (Cambridge, MA, USA). MAPK and phospho-MAPK family antibody sampler kits (#9926, #9910) were purchased from Cell Signaling Technology (Beverly, MA, USA); SB203580 (a p38 MAPK inhibitor) and Anisomycin (a p38 MAPK activator) were purchased from Calbiochem (San Diego, CA, USA) and Sigma-Aldrich respectively. Horseradish peroxidase (HRP)-conjugated anti-rabbit IgG, and anti-β-actin antibodies were purchased from Zhongshan Goldenbridge Biotechnology (Beijing, China). Alexa Fluor-594-conjugated secondary antibody was obtained from Invitrogen.

### Induction of diabetic mice

The Ethics Committee of Nanfang Hospital, Southern Medical University, approved all of the protocols and procedures using animals (approval number: NFEC-201112-K6). A total of 14 female C57BL/6J mice (6–8 weeks old, weighing about 20 g) were obtained from the Animal Center of Southern Medical University (Guangzhou, China). All were housed in specified pathogens-free conditions and fed standard rodent chow and fresh distilled water. After 1-week quarantine, mice were divided randomly into normal control (NC) group and DM group, composed of seven mice each. Diabetes was induced with streptozotocin (STZ, 40 mg/kg) injected into abdominal cavity for five consecutive days by the method previously reported 16. In NC group, mice were administrated with equal volume of citrate acid buffer as the solvent of STZ. All mice were killed at week 10 after modelling. Tissue samples removed from small intestines and serum samples were used to measure the expression of the interest proteins (Sdc1, HPSE, occludin, ZO-1, total/phosphorylated MAPK, *etc*.).

### Cell culture and *in vitro* diabetes models

Normal rat small intestine crypt cell line (intestinal epithelial cell 6, IEC-6) was obtained from American Type Culture Collection (Rockville, MD, USA) and was maintained in DMEM (Gibco, Cambridge, MA, USA) supplemented with 10% foetal bovine serum (Gibco) at 37°C with an atmosphere of 5% CO_2_. Cells were grown on polyester membranes in Transwell inserts (6.5 mm, pore size 0.4 μm; Costar, Cambridge, MA, USA), glass or culture plates to be the adequate model for further study. Cells were conventionally grown for 96 hrs (48 hrs for immunofluorescence assay) before subsequent stimulations. To assess DM model, IEC-6 cells were exposed to normal (NG, 12.5 mM) or high concentration of d-glucose (HG, 50 mM); the corresponding control groups were exposed to l-glucose (LG) in normal medium with d-glucose (12.5 mM d-glucose plus 37.5 mM l-glucose) to account for medium hyperosmolarity. In addition, to evaluate the therapeutic effect, IEC-6 cells were cultured in the presence of high glucose alone (50 mM d-glucose, for 24 hrs, NC group), or high glucose with insulin (0.01 unit/ml, for 24 hrs, Ins group), or with heparin (0.5 μg/ml, for 24 hrs, Hep group), or with SB203580 (10 μg/ml, for 90 min., MI group) 17,18. Culture supernatants and whole cell lysate were harvested at designated time and were stored for subsequent evaluations.

### Measurement of transepithelial electrical resistance

Exactly, 2.0 × 10^6^ IEC-6 cells per well were seeded on the collagen-coated membrane Transwell inserts with 200 μl culture medium added to the apical chamber and 600 μl to the basolateral chamber. The electrical resistance of confluent polarized IEC-6 monolayers was measured by transepithelial electrical resistance (TEER) with an electrical resistance system (EVOM; World Precision Instruments, Berlin, Germany). A pair of chopstick electrodes was placed at each of the apical and basolateral chambers of three different points to evaluate TEER. Readings were taken every 24 hrs until the net TEER had risen steadily above 250 Ω cm^2^ (at days 5–7). At this point, regulatory factors (PBS, d-/l-glucose, heparin, insulin, HPSE mAb, p38 MAPK inhibitors, *etc*.) were added to both the apical and the basolateral chamber, and TEER value was recorded at the appointed time after these interventions.

### ELISA

Target proteins in culture medium and serum were quantitative determined by ELISA kits according to the manufacturer's instructions. Briefly, samples, standards and diluted biotinylated antibody were added into precoated wells and incubated for 1 hr at room temperature. After 3 washes, horseradish peroxidase–streptavidin conjugate was added, and the plate was incubated for 30 min. at room temperature, then substrate was added and the colour was allowed to develop for 10–15 min. The reaction was stopped with sulphuric acid, and the absorbance was read at 450 nm in an ELISA plate reader (Bio-Rad, Hercules, CA, USA). The concentrations of target proteins were calculated based on the standard curve.

### Western blotting

Total cellular proteins were extracted and separated in 10% SDS-PAGE gels and then transferred to Polyvinylidenefluoride (PVDF) membrane. PVDF blots were blocked with a solution of 5% (w/v) skim milk in Tris-buffered saline containing 0.1% Tween-20 (TBS-T) for 1 hr at room temperature. The primary antibodies were diluted and added, and the blots were incubated overnight at 4°C. After washed in TBS-T, the blots were incubated in relevant secondary antibodies at a dilution of 1:5000 for 1 hr. Immunoreactive bands were visualized using Immobilon Western HRP Substrate (Merck Millipore, Billerica, MA, USA) and analysis with the Bio-Image Analysis System (Syngene, Frederick, MD, USA). Band intensity was normalized to β-actin and quantitated by densitometry using Image J software (National Institutes of health). Data represent the average of three separate experiments.

### Real-time PCR

Quantitative real-time PCR (qRT-PCR) was performed as described. Briefly, total cell RNA was prepared. Amplification reactions were performed in triplicate on Roche LightCycler 480 Real-Time PCR System. The primers used for mouse Sdc1 19 were 5′-TGG AGA ACA AGA CTT CAC CTT TG-3′ (forward) and 5′-CTC CCA GCA CTT CCT TCC T-3′ (reverse). The primers used for mouse HPSE were 5′-CAA GAA CAG CAC CTA CTC AAG-3′ (forward) and 5′-AGC AGT AGT CAA GGA GAA GC-3′ (reverse). Data were analysed using the 2^−ΔΔCt^ method with GAPDH as the constitutive marker.

### Immunofluorescence

Monolayers on chamber slides or plates were washed three times with PBS and fixed in 4% paraformaldehyde for 10–15 min. at room temperature. After being made permeable with 0.5% Triton X-100 in PBS for 5 min., cells were then blocked with 5% PBS-diluted bovine serum albumin (blocking solution) for 30 min. at room temperature, and were incubated with the interest primary antibodies overnight at 4°C. After washes with PBS, the monolayers were incubated with the Alexa fluor-594 conjugated antibody (1:500) at room temperature for 1 hr in the dark. Cells were washed with PBS and nuclei stained with DAPI (Invitrogen, Carlsbad, CA, USA) and examined under the Olympus BX51 microscope (Tokyo, Japan).

### Statistical analysis

All statistics were determined by SPSS software (Version 13.0, Chicago, IL, USA). Descriptive statistics were calculated with means and standard errors, then independent samples *t*-test for comparing means between two groups and one-way anova test for among three or more groups (with LSD method for *post hoc* multiple comparisons) were used. Comparisons of ranked data were determined by Mann–Whitney *U*-test (between two independent groups) or Kruskal–Wallis *H*-test (among multiple independent groups). Spearman's correlation analysis was employed to define associations. A *P* < 0.05 was considered significant.

## Results

### Dramatic Sdc1 destruction with synchronous HPSE elevation in diabetic mice

**Figure 1:**
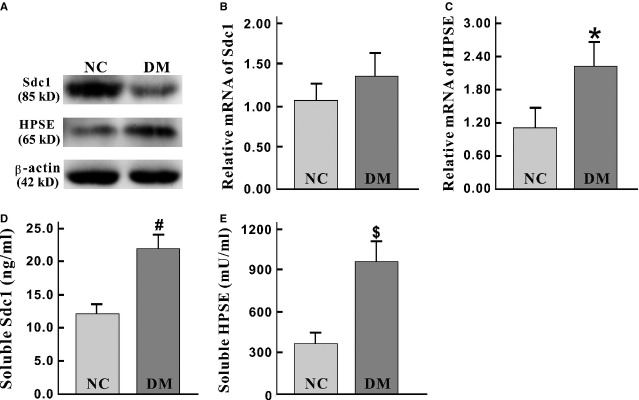
Significant alterations of tissue and serum Sdc1 and HPSE in diabetic mice. (A) Tissue expression of Sdc1, HPSE and β-actin in normal control (NC) and diabetic group (DM) were measured with Western blotting. Decreased Sdc1 and increased HPSE expression were shown in DM mice (*P-*value are 0.002, 0.003, respectively). β-actin was selected as an equal loading control. (B) No significant difference of the level of Sdc1 mRNA was found between NC and DM group (*P* = 0.159), suggesting the possibility of the abnormal degradation of Sdc1. (C) Compared to NC group, the level of HPSE mRNA significantly increased in DM group (*P = 0.009). (D and E) Serum Sdc1 (D) and HPSE (E) were measured with ELISA. Both Sdc1 and HPSE level were remarkably increased (^#^*P* = 4.97 × 10^−6^, ^$^*P* = 1.35 × 10^−5^). Sdc1: syndecan-1. HPSE: heparanase; Error bars: ±2.00 SE.

*In vivo* levels of Sdc1 and HPSE under control and diabetic conditions were detected by Western blotting, qRT-PCR and ELISA. In the diabetic group (DM), tissue Sdc1 from small-intestinal samples was significantly lower (1.002 ± 0.076 *versus* 0.510 ± 0.065, *P* = 0.002, Fig.1A), but qRT-PCR showed no significant changes of Sdc1 mRNA when compared with NC (1.062 ± 0.106 *versus* 1.350 ± 0.149, *P* = 0.159, Fig.1B), which indicated the decreases of Sdc1 protein derived from the destruction after its synthesis. Correspondingly, both of tissue protein and mRNA of HPSE were synchronously increased (0.184 ± 0.032 *versus* 0.402 ± 0.028, *P* = 0.003; 1.121 ± 0.172 *versus* 2.203 ± 0.236, *P* = 0.009) (Fig.1A and C). Meanwhile, the average of serum Sdc1 in NC and DM group were 12.2 ± 0.788 ng/ml and 22.2 ± 1.01 ng/ml (*P* = 4.97 × 10^−6^, Fig.1D), serum HPSE were 364.3 ± 43.3 mU/ml and 961.8 ± 73.0 mU/ml (*P* = 1.35 × 10^−5^, Fig.1E) respectively, both exhibiting notable elevations in DM groups. Significant correlations were found, not only between serum and tissue Sdc1 (*r* = −0.701, *P* = 0.005) but also between serum and tissue HPSE (*r* = −0.837, *P* = 1.86 × 10^−4^).

### The alterations of Sdc1 and HPSE under high-glucose cultivation

**Figure 2:**
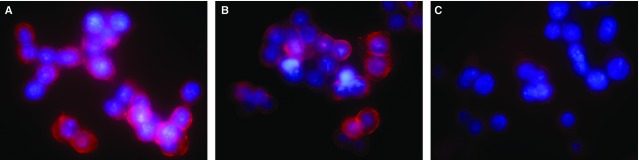
Sdc1 destruction significantly increases in high D-glucose culture medium. IEC-6 cells were cultured for 24 hrs in the presence of 12.5 mM D-glucose (A, NC group), 12.5 mM D-glucose plus 37.5 mM L-glucose (B, LG group) or 50.0 mM D-glucose (C, HG group). Immunofluorescence was applied as described in ‘Materials and methods’. Sdc1 was visualized by staining Alexa fluor-594-conjugated antibody (red) and nucleuses were stained with DAPI (blue). Original magnifications for all: ×400. Compared to the NC and LG group, the amount of cell surface Sdc1 significantly decreased in HG group.

**Figure 3:**
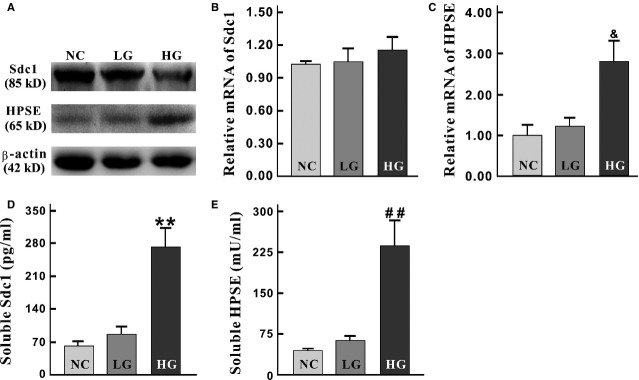
The alterations of Sdc1 and HPSE under high-glucose cultivation. (A) Western blots were applied with antibodies against Sdc1, HPSE and β-actin (as an equal loading control). Compared to the NC and LG group, the amount of Sdc1 significantly decreased in HG group (*P* = 0.027), and the expression of HPSE were increased (*P* = 0.027). (B) Cellular mRNA levels of Sdc1 was close among groups (*P* = 0.288). (C) The level of HPSE mRNA was significantly increased in HG group (*versus* NC and LG group, ^&^*P* = 0.039). (D and E) Soluble Sdc1 (D) and HPSE (E) were measured with ELISA. Both Sdc1 and HPSE level were remarkably increased (**P = 0.001, ^##^*P* = 0.009). Sdc1: syndecan-1. HPSE: heparanase; Error bars: ±2.00 SE.

IEC-6 cells cultured in high-glucose medium were used to further validate the alterations of Sdc1 and HPSE. Similar to the results obtained from diabetic mice, high glucose led to obvious Sdc1 destruction (Figs2A and C, 3A, B and D) and HPSE elevation (Fig.3A, C and E). But it is also worth noting that no statistical differences were observed between NC (with normal culture medium) and LG group (with l-glucose culture medium) (Figs2 and 3). These results also indicated the above alterations were not derived from the hyperosmolarity, but from some specific intracellular mediators and (/or) pathways after abnormal D-glucose absorption.

### The involvement of p38 MAPK in Sdc1–HPSE dysregulations

**Figure 4:**
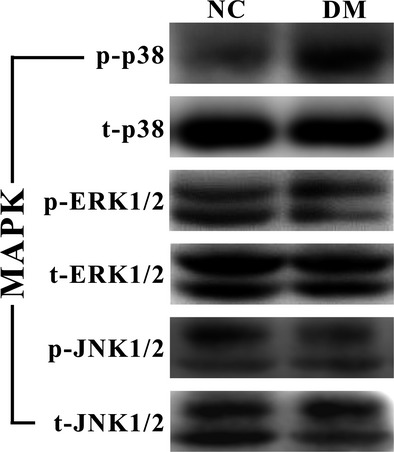
Diabetic condition increases the phosphorylation of p38 MAPK, but not ERK1/2 or JNK1/2. Western blots were probed with antibodies against phosphorylation-specific (p-) and total (t-) p38, ERK1/2 and JNK1/2. Compared to NC group, p/t-p38 was significantly increased in DM group (*P* = 0.002), while phospho-ERK and phospho-JNK showed no statistically significant changes (*P* = 0.141, 0.338, respectively). Statistically significant correlations were discovered between p/t-p38 and tissue Sdc1 (*r* = −0.785, *P* = 0.001), and p/t-p38 and tissue HPSE (*r* = 0.829, *P* = 2.5 × 10^−4^). NC: normal control group, DM: diabetic group. Molecular weight: p/t-p38, 40 kD; p/t-JNK1/2, 46 and 54 kD; ERK1/2, 42 and 44 kD.

Because high glucose activates the MAPK pathway in certain cells (mesothelial cells, neuroblastoma cells, *etc*.) 20,21, we then attempted to investigate MAPK activity in intestinal epithelial cells under diabetic/high-glucose condition and its possible involvements in Sdc1–HPSE dysregulations. Three main members of MAPK family, including p38, ERK and JNK, had been examined by Western blotting (Fig.4). Compared to NC mice group, the ratio of phospho-to-total p38 MAPK (p/t-p38) was significantly increased in DM mice group (0.202 ± 0.020 *versus* 0.427 ± 0.023, *P* = 0.002), but the ratio of phospho-to-total ERK1/2 (p/t-ERK1/2, 0.120 ± 0.008 *versus* 0.139 ± 0.009, *P* = 0.141) and phospho-to-total JNK1/2 (p/t-JNK1/2, *versus* 0.376 ± 0.016 *versus* 0.354 ± 0.015, *P* = 0.338) showed no statistical difference.

**Figure 5:**
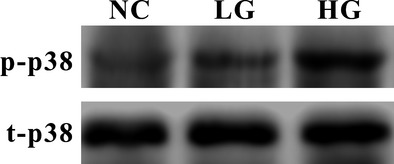
The activation of p38 MAPK under high-glucose cultivation. IEC-6 cells were cultured as mentioned in ‘Materials and methods’. Western blots were applied with antibodies against phosphorylation-specific (p-) and total (t-) p38. Compared to the NC and LG group, p/t-p38 MAPK of HG group was increased remarkably (*P* = 0.039). Notably, significant correlations were also showed between p/t-p38 and cellular Sdc1 protein (*r* = −0.867, *P* = 0.002), soluble Sdc1, HPSE protein and soluble HPSE (all *r* = 0.867, *P* = 0.002).

Similarly, the increased activity of p-p38 MAPK was observed in the cells after high concentration of d-glucose exposure (Fig.5, *P* = 0.039). Remarkably, the activation of p38 MAPK is strongly associated with the alternations of Sdc1 and HPSE (*P* < 0.05, Figs4 and 5). SB203580, a specific inhibitor of p38 MAPK pathway, was applied and ELISA assay showed that SB203580 significantly down-regulated soluble Sdc1 (Fig.8B, 255.2 ± 15.42 *versus* 180.2 ± 10.3 pg/ml, *P* = 4.1 × 10^−5^) and HPSE (Fig.8D, 197.6 ± 15.8 *versus* 140.4 ± 8.9 mU/ml, *P* = 0.0004) in HG culture medium. These indicates the possibility of enhanced p38 MAPK aggravates Sdc1 cleavage and HPSE overexpression in diabetic/high-glucose condition.

### Sdc1–HPSE and p38 MAPK synergistically exacerbate barrier destruction

**Figure 6:**
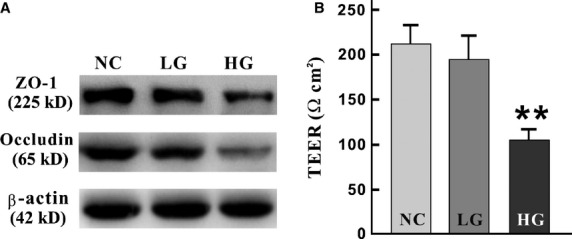
The negative regulation of tight junctions and TEER after high concentration of D-glucose exposure. IEC-6 cells were cultured as mentioned in ‘Materials and methods’. (A) Western blots were applied with antibodies against Occludin, ZO-1 and β-actin (as an equal loading control). The amount of Occludin was obviously decreased in HG group (*versus* NC and LG group, *P* = 0.039), while the overall contrast of ZO-1 was insignificant (*P* = 0.061). (B) Transepithelial electrical resistance (TEER), representing intestinal permeability and barrier functions, was measured by EVOM. Compared with NC and LG group, TEER dropped significantly in HG group (**P = 0.001). There were obvious correlations among TEER, Sdc1 and HPSE (between TEER and cellular Sdc1, HPSE expression, *P* = 0.042, 0.013, respectively; between TEER and soluble Sdc1, HPSE level, *P* = 0.049, 0.013, respectively).

Compared to NC and LG group, the relative expression of Occludin in HG group was remarkably decreased (χ^2^ = 6.489, *P* = 0.039, Fig.6A). Though the overall difference among groups was not statistically significant (χ^2^ = 5.600, *P* = 0.061), the relative expression of ZO-1 in HG group had shown much lower than NC and LG group (*versus* 0.220 ± 0.025 *versus* 0.802 ± 0.101, 0.647 ± 0.072) (Fig.6A). Meanwhile, the IEC-6 cell monolayers in high concentration of d-glucose, instead of normal concentration of d-glucose or isotonic l-glucose, had an obviously lower TEER values (104.7 ± 6.0 *versus* 212.1 ± 10.9 and 195.4 ± 12.8 Ω cm^2^, *P* = 0.001), indicating a serious damage to the barrier structure and function (Fig.6B). Specifically, correlation analyses suggested that there were apparently correlations among Sdc1, HPSE and TEER (all *P* < 0.05, Fig.6B).

**Figure 7:**
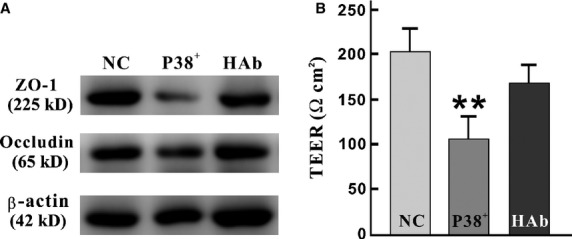
HPSE blockade attenuates barrier dysfunction induced by p38 MAPK activation. IEC-6 cells were cultured with 12.5 mM D-glucose (NC group), with p38 activator (Anisomycin, p38^+^ group), or with both Anisomycin and HPSE monoclonal antibody (HAb group). (A) The alterations of Occludin and ZO-1 were significant (both *P* were 0.027, χ^2^ = 7.200). Western blots were applied. (B) TEER dropped significantly after Anisomycin was added, but remarkably raise when co-incubation with Anisomycin and HPSE (**P = 0.004, *F* = 15.399).

HPSE blockade and p38 stimulation were applied to further investigate the interplay among p38, Sdc1/HPSE alteration and barrier disorder. Compared with normal culture conditions (with 12.5 mM d-glucose, NC group), the expressions of Occludin, ZO-1 (Fig.7A) and the level of TEER (Fig.7B) in cells treated with Anisomycin (p38 activator) were remarkably decreased. However, when Anisomycin and HPSE mAb were both added, dramatic reversals of the decreases of Occludin, ZO-1 and TEER were performed (Fig.7). These results indicated that under high-glucose condition, HPSE was essential and acted synergistically with p38 to trigger barrier destruction.

### The effects of insulin, Sdc1 analogue and p38 inhibitor on barrier functions

**Figure 8:**
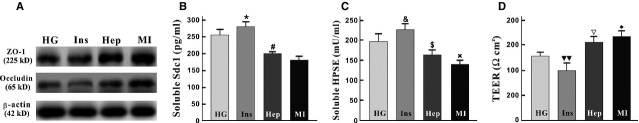
Treatments regulates Sdc1/HPSE expression and barrier function in high-glucose condition. IEC-6 cells were conventionally grown and processed with high glucose alone (HG group), or high glucose with insulin (Ins group), or high glucose with heparin (Hep group), or high glucose with SB203580 (MI group) as mentioned in ‘Materials and methods’. (A) The comparison of Occludin (*P* = 0.016) and ZO-1 (*P* = 0.025) in groups was significant. (B and C) Instead of insulin, heparin and SB203580 help alleviate the cleavage of Sdc1 (*^/#^*F* = 51.3, *P* = 1.4 × 10^−5^) and inhibit the elevation of HPSE (^&/$/x^*F* = 29.2, *P* = 1.2 × 10^−4^). (D) TEERs in Hep and MI group were obviously higher than those in HG and Ins group (^▾▾/▵/♦^*F* = 23.2, *P* = 2.7 × 10^−4^).

As high-glucose cultivation led to dysregulations of Sdc1/HPSE, p38 activation and barrier damage, we consequently investigated whether insulin uptake or interventions targeted at Sdc1/HPSE and p38 could provide therapeutic effect. As complements to the above results, it was found that insulin caused the increased cleavage of Sdc1 (HG *versus* Ins group, 255.2 ± 8.9 *versus* 281.4 ± 7.1 pg/ml, *P* = 0.022) and higher expression of HPSE (197.6 ± 9.1 *versus* 226.6 ± 7.4 mU/ml, *P* = 0.019) in HG culture supernatants, while heparin (Sdc1 analogue) effectively reduced both soluble Sdc1 (200.2 ± 4.6 pg/ml, *P* = 0.0003) and HPSE (164.2 ± 5.67 mU/ml, *P* = 0.01) (Fig.8B and C). The subsequent Western blot and TEER measurement showed that insulin could not relieve the reduction in Occludin and ZO-1 (Fig.8A); the co-incubation with insulin and high glucose even reduced TEER by about 26.8% than high glucose alone (Fig.8D; HG *versus* Ins group, *P* = 0.007). On the contrary, heparin and SB203580 effectively increased the expressions of Occludin and ZO-1, and TEER (*P* = 0.016, 0.025, 2.7 × 10^−4^, respectively; Fig.8A and D).

## Discussion

In the present study, dramatic destruction of Sdc1, elevation of HPSE expression, p38 MAPK activation and synchronous damage of intestinal barrier are performed after HG stimulation. These alterations are effectively overturned by exogenous additions of heparin (Sdc1 analogue) and SB203580 (p38 MAPK inhibitor), but not insulin. Our study therefore suggests the implications of Sdc1/HPSE abnormities, and therapeutical value of the relevant modulations towards intestinal barrier dysfunction in HG conditions.

Reduced Sdc1 and elevated HPSE have been commonly reported in malignant tumours, bacterial infections and chronic inflammatory diseases 13,22,23. The abnormities of Sdc1 and HPSE under HG conditions might provide an alternative perspective to better interpret HG induced/related enteropathy. For example, previous studies have showed Sdc1 participates in modulating extracellular growth factors binding/activities and relevant signalling pathways, the pathological loss of Sdc1 therefore partly accounts for abnormal proliferation and death/apoptosis raised in HG condition 9,24. Reduced Sdc1 and elevated HPSE presented in HG condition and after insulin treatment also offer some interpretations for the phenomenon that patients with type 2 diabetes have higher incidence of colorectal carcinoma, while insulin therapy may even increase this risk 25. Recently, studies have demonstrated that Sdc1 and HPSE are important endogenous regulators of energy balance and nutrient metabolism. HPSE overexpression and subsequent HS chains cleavage would not only cause diarrhoea, reduce reflex hyperphagia and food intake 26–28, but also alter lipoprotein lipase and clearance of triglyceride 29,30. These emphasize yet again the diverse functions of Sdc1 and HPSE, and suggest their pathological effects of Sdc1/HPSE imbalance on systemic and intestinal homoeostasis under HG condition.

The exact mechanism of Sdc1 alteration in HG group is not identified. Our study has showed that HG stimulation does not reduce Sdc1 mRNA level. In this view, HG stimulation should not been the direct effector of Sdc1 reduction and the decrease in Sdc1 protein arises from the destruction after its synthesis. Owing to the physiological functions and well correlation with Sdc1 alteration, enhanced HPSE should be one of the most essential components causing Sdc1 destruction. A relevant study conducted by Yang *et al*. 11 has also showed that HPSE regulates both the level and location of Sdc1 within the tumour microenvironment by enhancing its synthesis and subsequent shedding from the tumour cell surface. From this perspective, HPSE is an important target of HG stimulation.

The damages of TJs are still thought to be important manifestations of intestinal morphological and functional alterations 3. Our study, however, has showed Sdc1/HPSE can play a unique role on TJs expressions. ZO-1 and Occludin decrease synchronously with Sdc1 destruction triggered by HG stimulation, but increase after adding Sdc1–HPSE regulators (heparin, HPSE mAb). Nowadays, the interactions between Sdc1/HPSE and TJs have been paid increasing attention. Smith *et al*. has also reported increased Claudin-2 expression begins in early HIV-1 infection and is co-expressed with Sdc1 in the intestinal epithelium 31. These results highlight the capacity of Sdc1/HPSE on TJs regulation, which are indispensable in barrier formation and maintenance.

Increased p38 phosphorylation after HG stimulation might be essential for the abnormalities of many cellular processes 32. For example, p38 activation promotes the production of fibronectin by the mesangial cells, and facilitates epithelial to mesenchymal transdifferentiation 33. Notably, it is supposed that there are complex relationships between HPSE and p38 MAPK pathway. Previous studies have showed HPSE may enhance three main MAPK members (including p38, ERK and JNK, solely or simultaneously) by phosphorylation 34–36; certain pathophysiology processes induced by HPSE could be mediated after p38 regulation 37,38. In our study, however, the concurrent of HPSE up-regulation and sole p38 activation is a unique feature of HG induction and the mechanism for sole p38 activation is still obscure. Meanwhile, our study revealed p38 MAPK inhibitor reduced HPSE expression and the barrier destruction triggered by p38 activation was alleviated after HPSE blockade. Therefore, there is not only regulatory relationship but also mutual cooperation/interaction between HPSE and p38 MAPK.

Hypoglycaemic therapy remains to be the major therapeutical method reducing damage to intestinal epithelial cells and barrier. The features of Sdc1 and HPSE alterations, however, might provide a new rationale for therapeutic approach. Though studies showed HS mimetics/HPSE inhibitors (heparin, PI-88, SST001, *etc*.) fortify Sdc1 content of vascular endothelial cells and prevent the progression of diabetic angiopathy 39,40, there are few studies investigating whether they protect against HG-induced gastrointestinal damage. Our study here has presented heparin effectively maintains tight junctions and barrier function in HG condition. Therefore, preservation of Sdc1/HS on epithelial cells may be a strategy to prevent and treat DM-associated gastrointestinal complications. Deserve to note that HS mimetics, including heparin and modified heparin, would disturb or inhibit the coagulation cascade. Hypo-/non-anticoagulant heparin and anti-HPSE drugs may become promising options for clinical application.

In conclusion, we have found the unique alterations of Sdc1 and HPSE in HG condition, and confirmed their interactions with p38 MAPK activation and maintenance of intestinal barrier. The results indicate that Sdc1/HPSE modulation can be viewed as a potential treatment for relieving HG-induced gastrointestinal damage.
